# A Structural Model of the Genome Packaging Process in a Membrane-Containing Double Stranded DNA Virus

**DOI:** 10.1371/journal.pbio.1002024

**Published:** 2014-12-16

**Authors:** Chuan Hong, Hanna M. Oksanen, Xiangan Liu, Joanita Jakana, Dennis H. Bamford, Wah Chiu

**Affiliations:** 1 Graduate Program in Structural and Computational Biology and Molecular Biophysics, Baylor College of Medicine, Houston, Texas, United States of America; 2 Verna and Marrs McLean Department of Biochemistry and Molecular Biology, Baylor College of Medicine, Houston, Texas, United States of America; 3 Department of Biosciences and Institute of Biotechnology, University of Helsinki, Helsinki, Finland; Institut Pasteur, France

## Abstract

Two crucial steps in the virus life cycle are genome encapsidation to form an infective virion and genome exit to infect the next host cell. In most icosahedral double-stranded (ds) DNA viruses, the viral genome enters and exits the capsid through a unique vertex. Internal membrane-containing viruses possess additional complexity as the genome must be translocated through the viral membrane bilayer. Here, we report the structure of the genome packaging complex with a membrane conduit essential for viral genome encapsidation in the tailless icosahedral membrane-containing bacteriophage PRD1. We utilize single particle electron cryo-microscopy (cryo-EM) and symmetry-free image reconstruction to determine structures of PRD1 virion, procapsid, and packaging deficient mutant particles. At the unique vertex of PRD1, the packaging complex replaces the regular 5-fold structure and crosses the lipid bilayer. These structures reveal that the packaging ATPase P9 and the packaging efficiency factor P6 form a dodecameric portal complex external to the membrane moiety, surrounded by ten major capsid protein P3 trimers. The viral transmembrane density at the special vertex is assigned to be a hexamer of heterodimer of proteins P20 and P22. The hexamer functions as a membrane conduit for the DNA and as a nucleating site for the unique vertex assembly. Our structures show a conformational alteration in the lipid membrane after the P9 and P6 are recruited to the virion. The P8-genome complex is then packaged into the procapsid through the unique vertex while the genome terminal protein P8 functions as a valve that closes the channel once the genome is inside. Comparing mature virion, procapsid, and mutant particle structures led us to propose an assembly pathway for the genome packaging apparatus in the PRD1 virion.

## Abstract 2

Modeling how PRD1, a dsDNA membrane-containing virus, packages its genome using electron cryo-microscopy.

## Author Summary

The life cycle of a virus involves serial coordination of viral molecular machines. These machines facilitate functions such as membrane fusion and genome delivery during infection, and capsid formation and genome packaging during replication and shedding. Icosahedral dsDNA viruses use one genome-translocation machine for both genome delivery and packaging. The genome-translocation machine of the membrane-containing bacterial virus PRD1 is composed of four packaging protein species at a unique vertex. Because these proteins do not follow the dominating icosahedral symmetry of the viral capsid, the structure of this vertex has remained elusive. In this study, we localize the unique vertex in the virus from raw electron cryo-microscopy images of the virus. We show that the genome-packaging complex of PRD1 replaces the regular 5-fold structure at the unique vertex and contains a transmembrane conduit as a genome translocation channel. We extend our structural studies to the procapsid—a precursor of the virus—and three packaging mutant particles, allowing us to localize all individual protein species in the complex. Based on these structures, we propose a model of the molecular mechanism of assembly and packaging in the life cycle of the PRD1 virus.

## Introduction

The functional and structural knowledge of assembly principles of macromolecular complexes, in general, and viruses, in particular, have extended our understanding of viral capsid maturation and genome packaging processes. The model systems used are most often double-stranded DNA (dsDNA) viruses composed of only proteins and nucleic acids. Viruses with lipids possess additional complexity when exploring the mechanistic and structural properties of such fundamental functions.

The common mechanism for the genome encapsidation in icosahedral dsDNA viruses, including head-tailed phages, herpes, pox, and adenoviruses, involves a translocation of the viral DNA into a preformed procapsid by an ATP-driven reaction powered by the packaging complex localized at a single vertex [1]. This single vertex-portal complex operates in both genome delivery and packaging. A dodecameric connector at a 5-fold vertex provides a conduit for nucleic acid entry into the capsid [2]–[5]. It is also an assembly site for the transiently associated packaging NTPase powering DNA translocation [6]. The DNA packaging complex in tailless icosahedral dsDNA viruses with an internal membrane, such as bacteriophage PRD1, operates in a similar manner, but is driven by a virion associated ATPase [7],[8]. In PRD1, the ATPase P9 powers DNA packaging and has, in addition to the Walker A and B motifs, a conserved motif that may contribute to its anchoring to the membrane [7]. P9 also shares sequence similarity with several other putative viral packaging ATPases, implying that this packaging mechanism might be common among the internal membrane-containing viruses [7]. The only structural evidence for the packaging components of a tailless icosahedral virus with a membrane comes from the crystal structure of the archaeal *Sulfolobus* icosahedral virus 2 (STIV2) packaging ATPase, which shows that these ATPases belong to the FtsK-HerA superfamily of P-loop ATPases, having both cellular and viral members [9],[10]. However, how the packaging complex is connected to the virion and how it provides a conduit through the internal membrane remain unknown.

The discovery that bacterial virus PRD1 and human adenovirus have the same major capsid protein (MCP) fold and virion architecture led to the hypothesis that viruses infecting host cells belonging to different domains of life are related, even though they do not share any detectable sequence similarity [11],[12]. This finding has led to the structure-based classification of viruses, and accordingly it was also proposed that viruses fall into a relatively small number of structure based viral lineages [13],[14]. One of these lineages is represented by PRD1 and includes several other viruses such as adenovirus, bacteriophage PM2, vaccinia virus, *Paramecium bursaria* chlorella virus 1 (PBCV-1), archaeal *Sulfolobus* turreted icosahedral virus (STIV), and virophage Sputnik [15]–[21]. In addition, there are also similar viruses with two MCPs instead of one. The relation of these viruses to the double β-barrel MCP containing viruses has been recently discussed [22],[23]. All these viruses are thought to derive from a common ancestor preceding the separation of the three domains of cellular life [13],[24],[25].

Bacteriophage PRD1 is the best-studied viral system, where the virion possesses an internal membrane (Figure S1). The broad structural information on PRD1, down to atomic resolution, has provided insights into assembly principles of complex viruses [26]–[28]. The mature virion (∼66 MDa) is formed of at least 18 protein species of which ∼ten are membrane associated, constituting about half of the membrane mass [29],[30]. The external *pseudo-T* = 25 icosahedral capsid shell of PRD1 is composed of 720 copies of the MCP P3 (43.1 kDa) cemented together by 60 copies of minor coat protein P30 (9.1 kDa) (Figure S1A) [26],[31]. The MCP P3 has a canonical double jellyroll fold, which is conserved within the lineage of PRD1-like viruses [11],[26]. The viral membrane, which is selectively acquired from the host plasma membrane, has a higher phosphatidylglycerol/phosphatidylethanolamine (PG/PE) ratio than that of its host [32],[33]. In addition, the lipids in the viral membrane are asymmetrically distributed between the leaflets—PE and PG are enriched in the inner and outer leaflets, respectively, most probably due to the high membrane curvature imposed by the capsid [27],[33].

In PRD1, the regular 5-fold vertex (receptor binding vertex) consists of the membrane anchor protein P16 (12.6 kDa), penton base protein P31 (13.7 kDa), receptor recognition protein P2 (63.7 kDa), and spike protein P5 (34.2 kDa) [26],[31],[34]–[37]. Protein P2 initiates infection by attaching to the host cell receptor [35],[38]. However, unlike head-tailed bacteriophages in which the tail hub is used to penetrate the host cell envelope and provide a channel for genome delivery, PRD1 uses its internal membrane that transforms into a tail tube penetrating the capsid through an opening at the unique vertex and crossing the host cell envelope [38]–[40]. The structural transition of the membrane triggers the release of the other vertex complexes leading to the loss of interaction between the capsid and the underlying membrane and allowing the tube to be formed [39].

Among the 12 icosahedral vertices, PRD1 has one unique vertex responsible for the packaging of its linear 14,297 bp-long dsDNA genome, where the covalently 5′ end linked terminal proteins are necessary for genome packaging as well as for replication (dsDNA-P8 complex; P8 is a 29.6 kDa protein) [8],[41],[42]. The unique vertex consists of transmembrane proteins P20 (4.7 kDa) and P22 (5.5 kDa) as well as proteins P6 (17.6 kDa) and P9 (25.8 kDa), which were identified by genetic analyses and immuno electron microscopy (Figure S1B) [7],[43]–[45]. Previous experiments have shown that there are naturally occurring empty procapsids that lack protein P9 and are incompetent to package the genome [8]. The *in vitro* packaging system applied to different PRD1 packaging mutants showed that while P9 is the packaging ATPase, the packaging efficiency factor P6 participates in the process, most probably by having a role in the incorporation of P9 into the unique vertex [7],[8],[45]. To date, the unique vertex still remains structurally elusive, mainly due to technical difficulties in identifying non-icosahedral features in a highly symmetrical virus particle for cryo-EM structural determination.

In this study, we report the structure of a viral packaging complex with a membrane conduit using cryo-EM reconstruction without icosahedral symmetry imposition at 12 Å resolution. Using virus particles devoid of specific unique vertex protein species allowed us to define the structure of this DNA translocation conduit and propose an assembly pathway for this portal structure crossing both the protein shell and the underlying viral membrane layer.

## Results

### Cryo-EM Structure of Mature PRD1 Virion Reveals the Unique Packaging Vertex with a Membrane Conduit

Resolving non-icosahedrally organized features that are essential functional components in icosahedral viruses remains a challenge. Using algorithms specific to handling icosahedral objects in the multi-path simulated annealing (MPSA) software package [46],[47], several non-icosahedrally symmetric features in icosahedral viruses have been revealed, such as the tail organization in cyanophage P-SSP7 [47], the portal in herpes simplex virus 1 B-capsid [4], and the portal in enteric phage P22 procapsid [5] and mature virion [2],[48].

**Figure 1:**
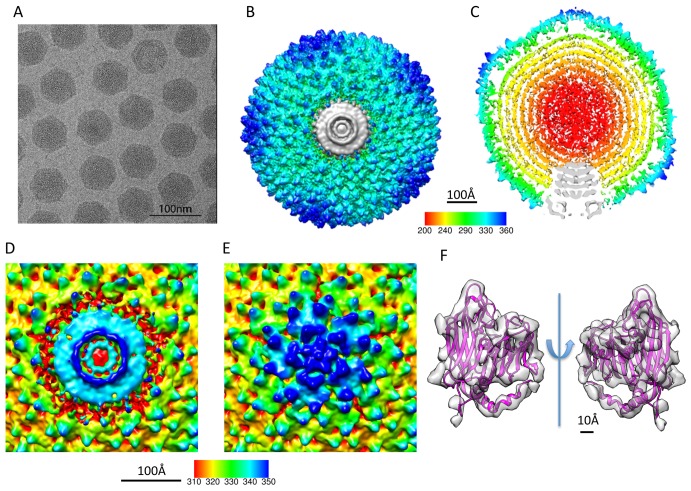
Reconstruction of PRD1 virion at 12 Å resolution without icosahedral symmetry imposition. (A) A typical micrograph of the PRD1 virions. (B) The top view and (C) the central slice view of the reconstruction. (D) The unique vertex occupies one of the 12 pentonal positions and interacts with the capsid proteins at its outer edge. (E) The other 11 vertices have a regular 5-fold structure. (F) The crystal structure of the MCP P3 of PRD1 (PDB code 1W8X, chain B) fitted in the corresponding segmented density in the cryo-EM map, allowing the boundary delineation of the unique vertex complex and the MCPs surrounding it.

In order to reveal the unique vertex in tailless mature PRD1 virion, 26,000 out of 50,000 particles were used to reconstruct the final density map at 12 Å resolution based on gold-standard criterion of two independent datasets [49],[50] without icosahedral symmetry imposition (Figures 1A–1C and S2A; Tables 1, 2, and S2; Movie S1). The map showed a unique packaging complex structure at one of its 12 vertices (Figure 1C and 1D) and regular 5-fold structures in the remaining 11 vertices (Figure 1E). The receptor recognition protein P2 and spike protein P5 were not resolved at the regular 5-fold vertices because of their flexible nature [37]. Except for the unique vertex, the overall virion density map revealed a similar capsid organization as in the X-ray structure of the icosahedral PRD1 capsid [26]. The Fourier shell correlation (FSC) calculated between the crystal structure of the MCP P3 (PDB: 1W8X, chain B) and the virion cryo-EM density map indicated that their structures match to 12 Å based on 0.5 FSC criterion (Figure S2B). This quantitative measure is substantiated by their apparent structural match (Figure 1F) and validates the overall accuracy of the image processing protocol.

**Table 1:** Wt and mutant PRD1 viruses and their properties.

Particle	Genotype	Mutant	Phenotype	Suppressor Strain^a^	Reference for the Particle	Complementation titers of PRD1 (pfu/ml)				Specific Infectivities of Purified Particles (pfu/mg of Protein)	
						Nonsuppressor, wt Strain DS88^b^	Suppressor Strain	Complementation Control Strain	Complementation Strain	Nonsuppressor, wt Strain DS88	Suppressor Strain
Virion	wt	—	wt	—	[82]	1.8×10^12^	—	—	—	1.4×10^13^	—
Procapsid	wt	—	P9*^−^*, DNA-P8*^−^*	—	[82]	—	—	—	—	2.2×10^11^	—
Sus621	*VI^−^*	*sus621*	P6*^−^*, P9^50%^, DNA-P8*^−^*	PSA	[45]	2.7×10^5^	7.3×10^10^	1.8×10^4^	3.4×10^9^	2.2×10^7^	3.0×10^10^
Sus526	*XX^−^*	*sus526*	P6*^−^*, P9*^−^*, P20*^−^* (P22^−^)^c^, DNA-P8*^−^*	DB7154	[44]	1.8×10^5^	1.8×10^11^	2.4×10^4^	7.1×10^6^	5.8×10^4^	4.4×10^7^
Sus42	*XXII^−^*	*sus42*	P6*^−^*, P9*^−^*, (P20*^−^*)^c^, P22*^−^*, DNA-P8*^−^*	PSA	[54],[58]	2.4×10^6^	4.3×10^11^	1.2×10^5^	5.2×10^9^	4.7×10^4^	6.4×10^7^

a *S. enterica* serovar Typhimurium LT2 suppressor strain harboring plasmid pLM2 [74],[75].

b *S. enterica* serovar Typhimurium LT2 DS88 [73].

c Presence of the protein in the particle is rather uncertain based on biochemical analysis.

**Table 2:** Properties of reconstructions of virion, procapsid, and mutant particles of PRD1.

Particle	Resolution of Reconstruction	Radius of the Capsid Shells^a^	Radius of the Inner Membrane^a^	Thickness of the Membrane^a^	Relative Membrane Intensity Compared to that of the Capsid Shell^b^
Virion	12 Å	283–322 Å	223–260 Å	37 Å	∼100%
Procapsid	14 Å	281–322 Å	200–245 Å	45 Å	∼80%
Sus621	19 Å	281–325 Å	198–243 Å	45 Å	∼75%
Sus526	22 Å	281–328 Å	203–240 Å	37 Å	∼69%
Sus42	18 Å	281–328 Å	203–240 Å	37 Å	∼62%

a Determined at full width half the maximum of the peak.

b Based on radially averaging the central section of each reconstruction and comparing the averaged intensity of the membrane layer to the capsid layer.

**Figure 2:**
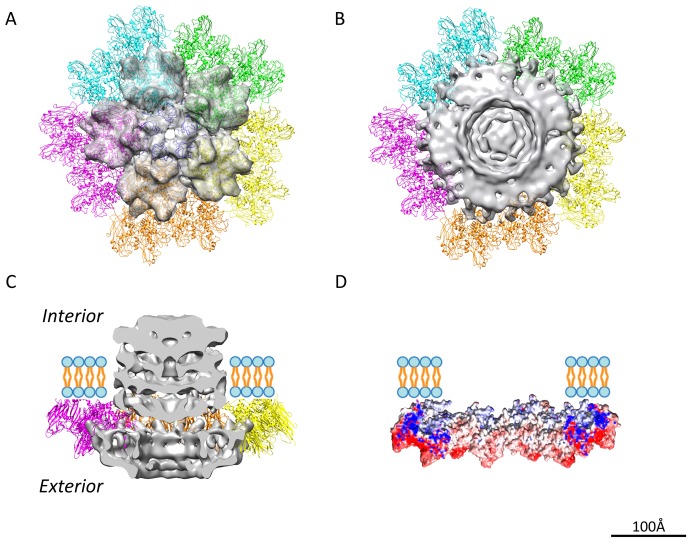
Delineating the boundary between the unique vertex and the surrounding major capsid proteins. (A) The ribbon representation of the regular 5-fold vertex structure (PDB code 1W8X). The penton P31 is shown in dark blue and the surrounding peripentonal P3 trimers are shown in five colors, each of which belongs to one asymmetric unit. (B) The top view and (C) the side slice view of the segmented unique vertex density (grey) surrounded by ten P3 trimers. The unique vertex replaces the penton and five peripentonal P3 trimers (shown as transparent gray density in (A)). (D) The electrostatic potential surface of the surrounding peripentonal MCP P3s was calculated with APBS and colored in Chimera ranging from red (negative) to blue (positive) with central slice view.

Crystal structures of the penton protein P31 and the MCP P3 [26] were docked into the cryo-EM density map (Figures 2A and 2B; Movie S2). The docking shows unambiguously that the unique vertex does not have the pentameric protein P31 and the five neighboring MCP P3 trimers (peripentonal MCPs) as do the regular 5-fold vertices (Figure 2A). At unique vertex position, the packaging complex is surrounded by ten MCP P3 trimers (Figure 2B). The segmented unique packaging vertex comprises not only the capsid region that replaces the regular 5-fold structure, but also the transmembrane region that anchors the inner membrane layer interior to the capsid shell (Figure 2C). To understand the interactions between the unique packaging vertex and the capsid shell, the electrostatic inner surface of the ten P3 trimers surrounding the packaging complex was calculated by APBS [51]. The inner surface of the surrounding MCPs had an overall weak negative charge, leading to a hypothesis that the outer surface of the packaging complex is positively charged to allow a stable interaction with the encompassing capsid shell (Figure 2D).

As we have determined the overall structure of the unique packaging complex in the mature virion, the locations of the four packaging protein candidates remain unassigned in the complex. We thus investigated the structures of the procapsid and three other packaging deficient mutant particles in order to localize the four protein species forming the packaging vertex.

### Cryo-EM Structures of PRD1 Procapsid and Sus621 Mutant Particle Reveal Only the Transmembrane Conduit Density at the Unique Vertex

Comparison of the mature virion to the procapsid devoid of packaging ATPase P9 and the viral genome (dsDNA-P8 complex) allowed the initial dissection of different protein components of the packaging vertex.

**Figure 3:**
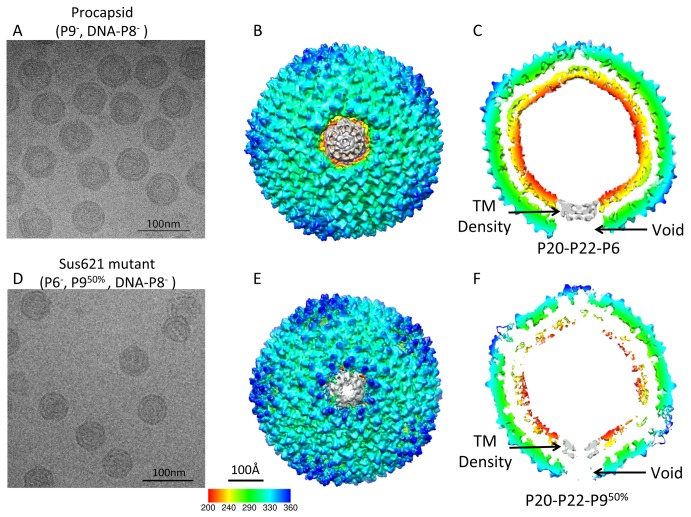
Reconstructions of the procapsid and Sus621 particle at 14 and 19 Å resolutions without icosahedral symmetry imposition. (A) Typical micrograph of the procapsid virus particles (P9*^−^*, DNA-P8*^−^*) containing P20, P22, and P6 without P9 and DNA-P8 complex at the unique vertex. (B) The top view and (C) the central slice view of the procapsid reconstruction. (D) Typical micrograph of the Sus621 virus particles (P6*^−^*, P9^50%^, DNA-P8*^−^*) containing P20, P22, and 50% P9 without P6 and DNA-P8 complex at the unique vertex. (E) The top view and (F) the central slice view of the Sus621 reconstruction. The unique vertex (grey) in either map only shows transmembrane densities but no ordered densities at the capsid region.

The procapsid density map without icosahedral symmetry imposition at 14 Å gold-standard resolution (Figures 3A–3C and S3A; Tables 1, 2, and S2; Movie S3) revealed that the organization of the MCP and internal lipid membrane was similar to that of the icosahedral map of the procapsid [28]. We noted a sharper fall-off of the FSC plot at low resolution between the two independent maps of the procapsid (Figure S3A) relative to that observed in the mature virion (Figure S2A), which can be attributable to disordering of the lipid membrane in the procapsid (Table 2). Docking of the crystal structure of MCP P3 into the symmetry-free procapsid density map (Figure S3B) revealed that their structures match. The FSC between the P3 crystal structure and the segmented P3 cryo-EM density shows a structural match to 14 Å based on the 0.5 FSC criterion (Figure S3C). On the basis of the difference map calculated between the procapsid and the mature virion at equivalent resolution (Figure S4A and S4B), the unique vertex of the procapsid displayed densities only in the transmembrane conduit but not at the radii of the capsid shell exterior to the membrane (Figure 3B and 3C). No density was observed on either side of the lipid bilayer confirming that protein P9 is part of the unique vertex. Since P9 is considered to reside at the external surface of the virus [8], we could attribute the missing density facing the exterior part of the virus to P9 (Figure 2C).

To localize the packaging efficiency factor P6 in the unique vertex, we utilized packaging deficient mutant Sus621 particles (amber mutation in gene *VI*), which are devoid of P6 and in which the amount of P9 is reduced to less than half of the wild-type (wt) amount (Table 1). The density map of the Sus621 particle at 19 Å gold-standard resolution (Figures 3D–3F and S5A; Table S2) revealed the transmembrane densities at the unique vertex similar to those seen in the procapsid (Figure 3B and 3C). The maps of the unique vertices in the procapsid and Sus621 particle lacked any density exterior to the membrane (Figure 3C and 3F). The icosahedrally arranged capsid proteins in the procapsid and Sus621 maps were structurally similar. However, the regular vertex penton densities showed higher structural variance in the Sus621 mutant particle than in the procapsid, as shown in their difference maps both compared against the mature virion map (Figure S4A–S4D). These suggest that the regular vertex pentons in the mutant particle are not as rigid as that of the mature and procapsid particles yielding higher variance in the reconstructed densities.

When examining closely at the transmembrane densities at the unique vertices of the procapsid and Sus621 maps (Figure 3C and 3F) and their difference map at the same resolution (Figure S6A), we found that there were extra densities in the center of the transmembrane densities in the procapsid map but not in the Sus621 map. Since protein P6 is present in the procapsid but not in the Sus621 particle, these additional densities may correspond to the region of the P6 anchored to the center of the transmembrane conduit, while the remaining region of the P6 exterior to the membrane is disordered in the absence of P9. Hydrophobicity cluster analysis of the P6 sequence reinforces the presence of hydrophobic domains within protein P6 (Figure S7A and S7B) [52],[53]. To explain these observations, we propose that the density exterior to the membrane at the unique vertex is a composite of P9 and portion of P6. The non-membrane region of protein P6 is disordered in the procapsid lacking P9, and protein P9 is disordered in the Sus621 particle in the absence of P6. When and only when P9 and P6 are both present, such as the case in the mature virion, they become well-ordered and their corresponding densities can be resolved (Figure 2C).

Furthermore, the rest of the membrane density in the Sus621 particle (Figure 3F) appears to be less pronounced than that of the procapsid and the mature virion (Table 2). This suggests that P6 may exert an impact on the membrane structure rigidity. The low resolution fall-off in the FSC curve of the two independent maps in the Sus621 mutant particle (Figure S5A) also supports this interpretation of the membrane disordering.

### Integral Membrane Proteins P20 and P22 Are Indispensable for the Formation of the Unique Vertex

In order to translocate the genome across the internal membrane of the virus, a transmembrane conduit has been proposed to exist at the unique vertex providing the channel for genome translocation [39]. Secondary structure element predictions by psipred [53] indicate that proteins P20 (4.7 kDa) and P22 (5.4 kDa) both have one long transmembrane helix and one short one (Figure S7C and S7D), implying that they can potentially form a transmembrane conduit at the unique vertex. To assign protein components to the transmembrane region of the unique vertex, two packaging deficient PRD1 mutants (amber mutation in gene *XX* or *XXII*) were exploited (Table 1). They are defective in the synthesis of protein P20 or P22, and form unpackaged particles also lacking proteins P6 and P9 (Sus526 and Sus42 particles) (Table 1). Based on biochemical analyses, it is not clear whether P20 and P22 are both simultaneously absent in the mutant particles [44],[54].

**Figure 4:**
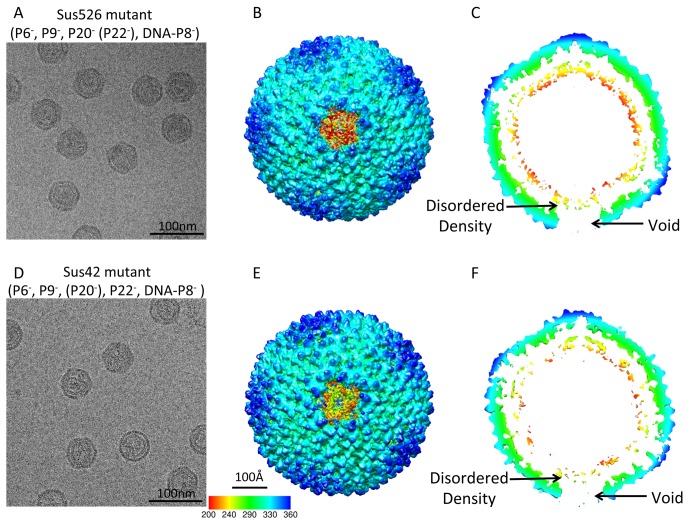
Reconstructions of two packaging vertex deficient particles at 22 and 18 Å resolutions without icosahedral symmetry imposition. (A) A typical micrograph of Sus526 mutant particles (P6*^−^*, P9*^−^*, P20*^−^* (P22*^−^*), DNA-P8*^−^*). (B) The top view and (C) the central slice view of the Sus526 reconstruction. (D) A typical micrographs of Sus42 mutant particles (P6*^−^*, P9*^−^*, (P20*^−^*), P22*^−^*, DNA-P8*^−^*). (E) The top view and (F) the central slice view of the Sus42 reconstruction.

In the cryo-EM images of Sus526 and Sus42 particles (Figure 4A and 4D), the membrane showed increased disorder and was unable to maintain a rigid shape. With a new non-icosahedral symmetry particle orientation search approach (details in Methods), we obtained the reconstructed density maps of the mutant particles determined at 22 Å and 18 Å gold-standard resolutions (Figure S5B and S5C; Table S2). This revealed a void density in the capsid region and a disordered density in the membrane region at the unique vertex (Figure 4B, 4C, 4E, and 4F), confirming that the unique vertex consists of proteins P6, P9, P20, and/or P22. Based on the difference map calculated at the same resolution between the Sus526 particle and the procapsid (Figure S6C and S6D) and that between the Sus42 particle and the procapsid (Figure S6E and S6F), the disordered transmembrane densities at the unique vertices of Sus526 and Sus42 particles do not contain the transmembrane conduit seen in the procapsid. In addition, the rest of the membrane density in Sus526 and Sus42 particles appears to be weaker than that of the mature virion and procapsid (Table 2). These observations suggest that proteins P20 and P22 contribute to the membrane density at the unique vertex and are critical to maintaining the integrity of the membrane. Without the presence of P20 and P22, the membrane exhibits additional flexibility.

### The Organization of the Unique Packaging Vertex

**Figure 5:**
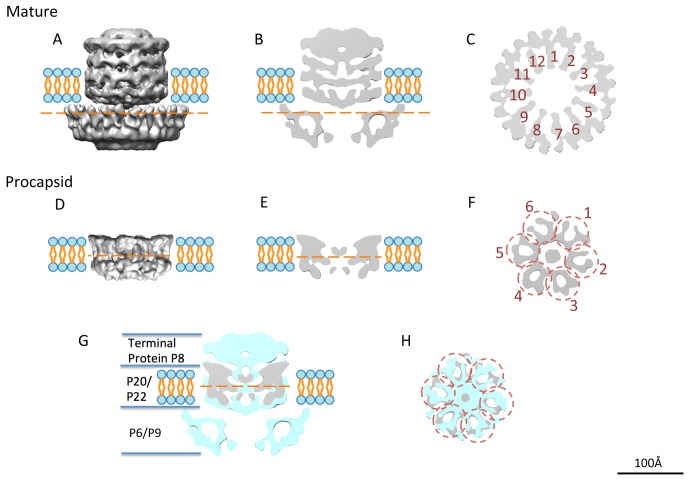
The unique vertex organization. (A) The side view, (B) the central slice view, and (C) the top slice view of the segmented unique vertex in the mature virion. The cut-through view (C) (location labeled as the orange dashed line in (A) and (B)) shows the 12 arms (numbered 1 to 12) of the central part of the unique vertex and the extra surrounding densities. (D) The side view, (E) the side slice view, and (F) the top slice view of the segmented transmembrane density in the procapsid. The top slice view (F) (location labeled as the orange dashed line in (D)) shows the 12 arms of the structure organized as hexameric dimers (numbered 1 to 6 in circles). The density of the unique vertex in the procapsid (grey) is shown on that of the mature virion (cyan) as a side slice view (G) and top slice view (H).

Following the localization of the four protein species in the unique vertex, we examined the detailed features of the segmented packaging complex from the mature virion (Figure 5A) and the transmembrane conduit from the procapsid (Figure 5D). Exterior to the membrane region at the unique vertex in the mature PRD1, the density is a composite of P6 and P9 and shows an apparent 12-fold symmetry based on rotational correlation curve (Figures 5C and S8A). It is surrounded by 10 MCP P3 trimers (Figure 2B and 2C). On the basis of the secondary structure prediction of P9 by psipred (Figure S7E) [53], the packaging ATPase P9 has a conserved α/β phage portal motif [55], suggesting that it can form a channel for genome translocation.

The density in the membrane region of the procapsid map displays an apparent 6-fold or 12-fold symmetry arrangement (Figure 5F). The volume of the transmembrane densities (excluding the central extra density that could belong to part of protein P6) is estimated to be around 83.6 nm^3^, which is equivalent to a molecular mass ∼69 kDa based on the previously established volume to mass equation [56]. Six copies of P20 and six copies of P22 add up to 60.6 kDa, which is reasonably close to the observed density. Based on rotational correlation curve (Figures 5F and S8B), the peaks at 6-fold symmetry arrangement were higher than at 12-fold one, also suggesting that the density is organized as a hexamer. Each of these hexameric components, potentially decorated by surrounding lipids, may represent a heterodimer made of one copy of P20 and P22. The central genome delivery channel, formed by P20 and P22, is estimated to be 40–50 Å wide. The assembly of P20/P22 complex may also provide the nucleating site for the packaging vertex assembly.

Interior to the membrane region, the packaging complex in the mature virion has an additional density, part of which probably corresponds to the terminal protein P8 complex with the dsDNA because this density is seen only in the virion (Figure 5G). A more close-up comparison of the density in the membrane region between the mature and procapsid maps showed some differences (Figure 5H), which may be caused by the membrane bilayer itself undergoing a conformational expansion between the states of procapsid and mature virion [28],[57]. The structural change of the membrane may as well be induced by the addition of P9, P6, and DNA-P8 onto the packaging complex.

## Discussion

### An Elegantly Built Packaging Motor with a Transmembrane Conduit

Many biological processes involve the utilization of ATP as the fuel source. One exemplary illustration of the extensive roles of ATPases is the encapsidation of viral genomic material into a preformed procapsid shell. PRD1 ATPase P9 provides the energy for the viral genome packaging as shown using an *in vitro* packaging assay [7],[8]. P9 has a dual role. Functionally, it is a powerhouse to fuel the packaging process by hydrolyzing ATP, and structurally, P9 with the packaging efficiency factor P6 form the portal providing the external part of the channel at the unique vertex for DNA translocation. The internal part of the packaging complex (P20/P22) at the unique vertex is embedded in the membrane, and provides the transmembrane conduit. P20/P22 complex also serves as the nucleating site for the whole specific vertex assembly.

### Symmetry Mismatches between the Protein/Protein Complexes at the Special Vertex

The MCP P3 of PRD1 forms an icosahedral shell with a *pseudo-T* = 25 lattice [11],[26]. At the regular 5-fold vertex, five P31 proteins organized as a penton with a strict 5-fold symmetry (Figure 2A). In the reconstruction without icosahedral symmetry imposition (Figures 1 and 2), there are 705 (720−5×3) P3 and 55 (60−5) P31 molecules forming the PRD1 capsid shell. The special vertex occupied by several protein components does not obey 5-fold symmetry (Figure 2B). Ten MCP P3 trimers wrap around the 12-fold symmetrical P9/P6 complex at the unique vertex (Figures 2B and 5A–5C). Such a symmetry mismatch is a structural hallmark of the head-tailed dsDNA viruses with a portal complex arranged with 12-fold symmetry [3],[4],[47],[48]. In PRD1 mature virion, the internal membrane follows the shape of the icosahedral capsid shell. This is presumably due to the pressure formed by the packaged genome, the presence of various membrane proteins, and the intercalation of the P3 shell into lipid moieties (Figure S1B). However, at the unique vertex, the proteins connected to the P9/P6 complex are membrane proteins P20 and P22, which are organized with 6-fold symmetry (Figure 5F). A 12-fold versus 6-fold symmetry mismatch among different components at the unique portal vertex, seen here at the interface between P20/P22 and P9/P6, is also found in membrane-less head-tailed dsDNA phages [47].

### Molecular Mechanism of Assembly and Packaging in the Life Cycle of PRD1

**Figure 6:**
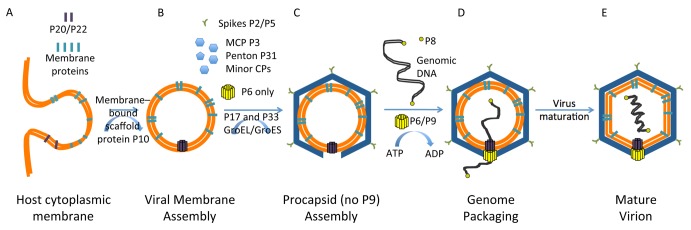
Proposed model for PRD1 procapsid assembly, genome packaging and virus maturation. (A) Viral membrane-associated proteins are incorporated into the host cytoplasmic membrane, including integral membrane proteins P20 and P22. (B) P20 and P22 form the transmembrane pore in the newly-assembled viral membrane. (C) Various capsid-associated proteins then assemble along the viral membrane to form the viral capsid, while P6/P9 assembles onto the transmembrane pore (P20/P22) and forms the unique packaging complex, which completes the assembly of the procapsid. (D) The newly synthesized viral genomic DNA is then packaged through the unique vertex fueled by the hydrolysis of ATP. (E) Along the genome packaging, the membrane is pushed against the icosahedral protein shell as the last step in the virus maturation.

We propose a molecular model for procapsid assembly and genome packaging (Figure 6), which will serve beyond PRD1 and provide one of the first structural clues in understanding the life cycle of the tailless internal membrane-containing icosahedral dsDNA viruses. As the first step, newly-synthesized viral membrane proteins are transported to the cytoplasmic membrane of the host cell (Figure 6A) [58]. The virus-specific membrane patch is then presumably pinched off, resembling the mechanism of the eukaryotic clathrin-coated pits, providing the framework for procapsid assembly (Figure 6B). The correct folding of certain viral structural proteins (e.g., MCP P3) and the formation of the PRD1 procapsid is facilitated by the host GroEL-GroES chaperonin and virus-encoded scaffolding protein P10 and assembly factor P17 (and most probably P33) [59]–[61]. Interestingly, procapsids devoid of the unique vertex can still assemble, which suggests that the membrane and/or other membrane proteins, for example, the membrane associated non-structural protein P10 [31], are functioning as a scaffold for capsid formation without the packaging complex. However, lacking the P20/P22 membrane pore in the unique vertex leads to disordered internal membrane layers as suggested by the weaker intensities in the membrane region of the map (Figure 4; Table 2). Since P20/P22 membrane pore is one of the defining features of the unique vertex, its absence leads to the formation of non-biologically active particles. In these particles, the specific interactions between the capsid shell proteins and the membrane could be altered, which would result in a weaker density in the map.

In the procapsid, in which P9 is absent, and the Sus621 particle lacking P6 and half of P9, the packaging process is deficient, but the presence of the P20/P22 conduit defines the unique vertex and thus allows the stable interactions between the capsid shell and the underlying membrane, making the internal membrane rigid (Figure 3; Table 2). However, without the internal genome pressure, certain flexibility may exist in the membrane envelop of the procapsid and Sus621 particle. Integral membrane proteins P20 and P22, which tend to form hexameric heterodimers (Figure 5F) with potential lipid decorations, assemble to form a transmembrane conduit (Figure 6C). Then, P9 and the packaging efficiency factor P6 form a 12-fold portal complex with P6 positioned atop the transmembrane conduit. P8 is linked to the 5′ end of the linear dsDNA genome and may recruit the genome to the packaging motor by binding to P9. After this complex is formed, the genome and genome-associated P8 begin to be packaged [45].

Once the packaging efficiency factor P6 and packaging ATPase P9 together become ordered in their position in the unique vertex, DNA packaging can begin. ATP hydrolysis by P9 provides the energy for DNA translocation into the procapsid through the unique vertex (Figure 6D). The conduit across the membrane formed by integral membrane proteins P20 and P22 provides the 40–50 Å wide channel for the dsDNA-P8 complex to be transported through the inner membrane underneath the capsid shell. After packaging, the pore in the vertex must be sealed. The terminal protein P8 may play a role as a protein valve similar to the valve of the head-tailed phage P-SSP7 [47].

After packaging the 14.9 kb dsDNA genome, the increased internal pressure leads to the expansion of the membrane, which dissipates the energy and prevents the massive expansion of the capsid shell. The mature PRD1 virion, as observed in this study, has undergone membrane expansion (Figures 1C and 3C; Table 2). The spacing of the lipid bilayer decreases upon the maturation of the particle and the membrane layer gets closer to the capsid shell as seen in our symmetry-free reconstructions as well as in the previous icosahedral maps (Table 2) [57],[62]. In addition, the internal genome pressure and the closer interaction between the membrane and the capsid shell make the membrane envelope most secure in its relative position and thus result in a stronger density in the reconstructed map (Table 2). These observed changes accommodate the packaging process and eventually lead to the maturation of PRD1 procapsid into infectious virion (Figure 6E).

### Comparison to Other Viral Packaging Pathways Utilizing Empty Proheads

Biochemical and structural analyses of the unique vertices in the head-tailed dsDNA bacteriophages such as T4, T7, ϕ29, P22, epsilon15, P-SSP7, and some eukaryotic viruses have demonstrated that their packaging and assembly processes share similarity both functionally and structurally. One example is the well-studied bacteriophage P22 [2],[5],[48], where the portal proteins function as the nucleating site for the procapsid assembly with the help of scaffolding proteins. Once the procapsid is formed, the DNA is packaged through the channel of the portal powered by the terminase motor with ATPase activity [63]. Virus maturation involves the release of the scaffolding proteins and terminase [64], before the tail is attached at the unique vertex.

In phage ϕ29 [65], the MCPs, connector/portal protein, head fiber proteins, and packaging RNA (pRNA) molecules together form the prohead with the help of the scaffolding proteins. Then the ATPase motor of ϕ29 packages the DNA into the prohead through the channel provided by the portal proteins and the pRNAs at the unique vertex. After that the tail is attached onto the unique vertex completing the assembly of the virion.

The assembly of PRD1 differs from the head-tailed viruses. First, PRD1 does not possess a conventional portal protein like the portal of phage P22 [66] or the connector in ϕ29 [67]. How is the PRD1 procapsid formed? The portal protein complex of P9 and P6 are assembled to the procapsid, providing the channel for DNA translocation. The ATPase activity of P9 provides the energy for DNA packaging [7] analogous to the terminase in P22 [68] or the ATPase in ϕ29 [69]. However, PRD1 P9 does not dissociate from the capsid after the DNA is packaged as in P22 and ϕ29 systems. Second, the packaging efficiency factor P6 of PRD1 serves as a facilitator for the ATPase motor in genome packaging. In contrast, the DNA-packaging motor of bacteriophage ϕ29 is geared by a ring of pRNAs [70]. Third, our study provides the structural insights into the packaging and assembly process in an icosahedral virus with an internal membrane. In this membranous virus, P20 and P22 form a transmembrane nanotube and provide a nucleating site for the recruitment of P9 and P6. For comparison, in the head-tailed bacteriophages like P22, the portal complex is the initiating site for procapsid assembly [5]. Finally, during maturation of the head-tailed dsDNA bacteriophages, such as HK97 [71] or P22 [5], the viral capsid goes through significant conformational changes including capsid expansion and angularization. In contrast, virus maturation in PRD1 mainly involves the membrane expansion and conformational changes at the MCP-membrane interface as well as in the transmembrane densities at the unique vertex without major conformational changes in the viral capsid [57],[62]. Thus, it is the inner membrane in PRD1 that undergoes most of the significant structural re-arrangements during virus maturation, not the viral capsid shell.

The unique vertex of PRD1 resolved here portrays the detailed structural picture to advance our understanding on procapsid assembly and genome packaging in a membrane-containing virus. The number of different PRD1-like icosahedral internal membrane-containing viruses is increasing: these can infect archaea, bacteria, and eukaryotes, covering all domains of life [72]. This is the first time, to our knowledge, that such a packaging-portal complex structure is revealed. Based on sequence data, all PRD1-like viruses encode a packaging ATPase, including the Walker A and B motifs and the P9-specific region [7] like PRD1 P9. However, even within this group of viruses the packaging mechanisms must differ between those with a circular or linear genome. For viruses with a linear genome, the packaging mechanism resembles that of PRD1, but for circular genomes, like in bacteriophage PM2, the mechanism for the packaging/condensation of the genome could be totally different [16].

## Materials and Methods

### Virus Production and Purification

Wt PRD1 and its packaging deficient mutants (Table 1) were propagated (LB medium at 37°C) on *Salmonella enterica* serovar Typhimurium LT2 DS88 (wt non-suppressor host) [73] and on *S. enterica* suppressor strain PSA (*supE*) [74] or DB7154 (*supD10*) [75] harboring plasmid pLM2. The suppressor-sensitive mutant phenotypes were verified by an *in vivo* complementation assay using plasmids carrying the corresponding PRD1 wt genes (Tables 1 and S1).

To reduce the background in mutant virus productions, the infected cells (multiplicity of infection 8) were collected 15 minutes post infection (Sorvall SLA3000 rotor, 5,000 rpm, 10 min, 22°C) and resuspended in pre-warmed fresh medium. Released virus particles were concentrated and purified by polyethylene glycol-NaCl precipitation, rate zonal (5%–20% gradient; Sorvall rotor AH629, 24,000 rpm, 55 min, 15°C), and equilibrium (20%–70% gradient; Sorvall rotor AH629, 24,000 rpm, 16 h, 15°C) centrifugations in sucrose using 20 mM potassium phosphate (pH 7.2), 1 mM MgCl_2_ buffer [76]. The equilibrated particles were concentrated by differential centrifugation (Sorvall rotor T647.5, 32,000 rpm, 2 h, 5°C) and resuspended in the same buffer. The protein concentrations were measured by Coomassie blue method using bovine serum albumin as a standard [77]. The wt/revertant backgrounds of the purified mutant particles were analyzed by assaying their specific infectivity on suppressor and wt hosts (Table 1). The protein pattern of the purified particles was analyzed by sodium dodecyl sulfate-polyacrylamide (16% acrylamide) gel electrophoresis (SDS-PAGE) [78].

### Cryo-electron Microscopy Data Acquisition

Aliquots of 2.5–3 µl of purified PRD1 particle suspension (Table 1) were applied to 400 mesh R1.2/1.3 Quantifoil grids (Quantifoil Micro Tools GmbH), blotted for 2 s and immediately frozen in liquid ethane using an automated vitrification device: either a Vitrobot MarkIII (FEI) or a Cryo-Plunger 3 (Gatan). Images were taken with a 300 kV JEM3200FSC electron microscope (JEOL) equipped with in-column energy filter. A slit width of 20 eV was used for data collection. The first dataset of the virion and all procapsid data was recorded at 80 K×nominal magnification (1.42 Å/pixel sampling) with a dose of 20 e/Å^2^ using a Ultrascan 4000 CCD camera (Gatan) with defocus ranging from 0.5 to ∼2 µm (Table S2). The second dataset of virion was collected using a Ultrascan 10000 CCD camera (Gatan) binned by 2 (1.3 Å/pixel sampling) with a defocus range from 1 to 3 µm. All mutant particles were imaged on a 200 kV JEM2010F electron microscope (JEOL) with a dose of 25 e•Å^−2^ using a Ultrascan 4000 CCD camera (Gatan) at 40–60 k×nominal magnification sampling from 1.81 to 2.18 Å/pixel and defocus ranging from 1.5 to 3 µm (Table S2).

### Cryo-EM Reconstructions

The virus particle images of PRD1 virion and procapsid were picked automatically with program ETHAN [79] and then manually screened using the EMAN2 program *e2boxer.py* [80]. Contrast transfer function (CTF) parameters of these particles were adjusted and determined using the EMAN program *ctfit* with detectable signals to ∼1/6 Å^−1^ in their 1D power spectra. The MPSA package was used to determine the icosahedral orientation of each particle starting from a random spherical model and only considering information below 10 Å to avoid model bias and over-fitting of the noise [46]. To exclude bad or low quality particles, the consistency of alignment parameters (both the orientation and the center) was used as the selection criterion. MPSA determines five orientation parameters simultaneously using Monte Carlo scheme for icosahedral and symmetry free virus reconstructions. If a raw particle is bad or low quality at a given resolution search range, the program would not yield a stable set of alignment parameters if repeating the orientation search multiple times. We used a strict consistency criterion (orientation difference <0.5°, center difference <3 Å) to compute the best possible icosahedral reconstruction. These criteria of particles selection were published in our earlier paper [46] and have been applied for many applications [4],[5],[47]. Using this algorithm, we filtered about 48% of the particle in our mature virion dataset and ∼20% in the procapsid and other mutants dataset. A possible reason of such a high rejection rate in the mature virion dataset could be the structural plasticity of the samples, which would be a more significant issue when reaching for higher resolution. EMAN *make3d* program was used to reconstruct the 3D map [81].

The algorithm for breaking the icosahedral symmetry and obtaining the asymmetric particle orientation has been previously described (Figure S9) [47]. Briefly, an initial icosahedral orientation was determined and an icosahedral reconstruction was obtained. Using a very low density threshold, a faint feature at the vertex was segmented out and used as a starting point to determine the asymmetric orientation. Through an iterative process, the features of the unique vertex were improved, which further allowed the more accurate assignment of the genuine asymmetric orientations out of the 60 equivalent possible choices (12 vertex locations×5 possible attachments of the symmetry mismatch at a 5-fold).

The selected particles were split to even and odd half-datasets at the beginning of the refinement (Table S2). Therefore, each half dataset was refined independently starting from separate random spherical models. The resolutions of the FSCs were calculated between two independent reconstructions without any masking for each virus particle dataset. The resolutions of the maps at 0.143 criterion were 12 Å for the wt virion (Figure S2A), 14 Å for the procapsid (Figure S3A), and 19 Å for the Sus621 particle (Figure S5A).

For the Sus526 and Sus42 mutant particles, the same algorithm and approach were attempted but no unique vertex complex was seen in either case. In order to find the missing unique vertex, a new algorithm was developed using the 11 regular 5-fold vertices as a reference for asymmetric search. For this approach, the Sus526 and Sus42 particles were oriented with best matches of regular 5-fold vertices and the missing unique vertex, if there is one, will be seen at the one remaining vertex location. This approach allowed us to successfully identify the orientation of the particles without the unique vertex. The independent FSCs resolution assessments were also done for these two maps, revealing the resolutions to be 22 Å for Sus526 particle and 18 Å for Sus42 particle (Figure S5B and S5C).

In order to compare maps of the virion, the procapsid and packaging mutants at various resolutions, difference maps were calculated between the two maps filtered at the same resolution (Figures S4 and S6). In each pair of comparison, the higher resolution map was filtered to the same resolution to the lower resolution map. The difference map between any two maps at same resolution was computed in Chimera with operation: *vop map1 subtract map2*. The difference map was displayed with surface color.

The even-odd FSC curves of the procapsid and packaging mutant particles (Figures S3A and S5) showed a moderate drop at the low-resolution region, which was not the case in that of the mature virion (Figure S2A). This observation could be caused by the fact that the membranes in the procapsid and packaging mutant particles are not as rigid as that in the mature virion where the genome pushed the membrane to secure its stable shape. Thus, the less rigid membrane could be one of the reasons for the moderate drop at lower resolution in the FSCs of procapsid and packaging mutant particles. When comparing only the density map of the MCPs in the procapsid to the corresponding X-ray structure (Figure S3C), such drop was not present in the FSC.

## Supporting Information

Figure S1 **Schematic illustration of PRD1 virion.** (A) PRD1 capsomer organization (PDB 1W8X) visualized by Chimera. Four MCP P3 trimers forming the asymmetric unit are colored in green, light blue, blue, and yellow. The penton protein P31 at the vertices is in red. (B) A schematic presentation of the PRD1 virion and functions of the virion proteins. Numbers in the parenthesis identify the corresponding protein structures in the Protein Data Bank.
(TIF)

Figure S2 **Resolution assessment for reconstruction of the virion without icosahedral symmetry imposition.** (A) FSC curve of gold-standard resolution test of the reconstruction without icosahedral symmetry imposition and without masking reveals the resolution to be 12 Å at 0.143 criterion. (B) Calculated FSC curve between the X-ray structure of P3 (PDB code: 1W8X, chain B) to the segmented density of P3 from our cryo-EM map reveals the resolution to be 12 Å at 0.5 criterion.
(TIF)

Figure S3 **Resolution assessment for reconstruction of the procapsid without icosahedral symmetry imposition.** (A) FSC curve of gold-standard resolution test of the reconstruction without icosahedral symmetry imposition and without masking reveals the resolution to be 14 Å at 0.143 criterion. (B) Segmented density of P3 chain B from the cryo-EM map of the procapsid docking with the corresponding crystal structure (PDB code: 1W8X, chain B). (C) Calculated FSC curve between the X-ray structure of P3 to our cryo-EM map reveals the resolution to be 14 Å at 0.5 criterion.
(TIF)

Figure S4 **Difference maps of procapsid (P9^−^, DNA-P8^−^) and Sus621 mutant particle (P6^−^, P9^50%^, DNA-P8^−^) compared to the mature virion.** (A) The side view and (B) the central cutaway view of the difference map between the procapsid and the virion filtered to the same 14 Å resolution. (C) The side view and (D) the central cutaway view of the difference map between the Sus621 mutant particle and the virion filtered to the same 19 Å resolution.
(TIF)

Figure S5 **Resolution assessments for the reconstruction of the Sus621, Sus526, and Sus42 mutant particles without icosahedral symmetry imposition.** (A) FSC curves of gold-standard resolution test of the Sus621 particle reconstruction without icosahedral symmetry imposition and without masking reveals the resolution to be 19 Å at 0.143 criterion. (B) FSC curves of gold-standard resolution test of the Sus526 particle reconstruction without icosahedral symmetry imposition and without masking reveals the resolution to be 22 Å at 0.143 criterion. (C) FSC curves of gold-standard resolution test of the Sus42 particle reconstruction without icosahedral symmetry imposition and without masking reveals the resolution to be 18 Å at 0.143 criterion.
(TIF)

Figure S6 **Difference maps of the Sus621 (P6^−^, P9^50%^, DNA-P8^−^), Sus526 (P6^−^, P9^−^, P20^−^ [P22^−^], DNA-P8^−^) and Sus42 (P6^−^, P9^−^, [P20^−^], P22^−^, DNA-P8^−^) particles to the procapsid (P9^−^, DNA-P8^−^).** (A) The side view and (B) the central cutaway view of the difference map between the Sus621 and the procapsid filtered to the same 19 Å resolution. (C) The side view and (D) the central cutaway view of the difference map between the Sus526 particle and the procapsid filtered to the same 22 Å resolution. (E) The side view and (F) the central cutaway view of the difference map between the Sus42 particle and the procapsid filtered to the same 18 Å resolution.
(TIF)

Figure S7 **Secondary structural elements predictions and hydrophobicity cluster analysis.** (A) Secondary structural elements (SSEs) prediction by psipred for P6. (B) Hydrophobicity cluster analysis by HCA for P6. (C) SSEs prediction for P20. (D) SSEs prediction for P22. (E) SSEs prediction for P9.
(TIF)

Figure S8 **Rotational correlation analysis.** (A) Rotational correlation curve of the unique vertex in the mature virus and (B) Rotational correlation curve of the transmembrane densities in the procapsid.
(TIF)

Figure S9 **An illustration for the workflow of virus reconstruction without icosahedral symmetry imposition.** Using the raw particle images, the icosahedral orientations were first determined to reconstruct the icosahedral map. An initial vertex volume (in the red cubic area) was extracted from the icosahedral map and made into a 3-D initial model as a mask of a putative portal. This initial 3-D portal mask was used to generate a library of 2-D masks corresponding to 60 possible locations for each raw particle image with known icosahedral orientation. 60 corresponding masked areas were extracted from each particle image and compared using cross common lines to the projections of the initial mask model. The best match among all the comparisons represents the most likely location of the unique vertex. The orientation that corresponds to the best match in the search for the unique vertex is the true asymmetric orientation for the particle. 3-D map of the virus would be reconstructed from the particles with true asymmetric orientations without imposing any symmetry. This process was iterated until the map had converged and no improvements could be seen.
(TIF)

Table S1 ***Escherichia coli*** **K12 HMS174 strains and plasmids used in the complementation assay.**
(DOCX)

Table S2 **Data collection of the virion, the procapsid, and three packaging mutant particles.**
(DOCX)

Data S1 **Numerical data for the FSC plot in Figure S2A.**
(XLSX)

Data S2 **Numerical data for the FSC plot in Figure S2B.**
(XLSX)

Data S3 **Numerical data for the FSC plot in Figure S3A.**
(XLSX)

Data S4 **Numerical data for the FSC plot in Figure S3C.**
(XLSX)

Data S5 **Numerical data for the FSC plot in Figure S5A.**
(XLSX)

Data S6 **Numerical data for the FSC plot in Figure S5B.**
(XLSX)

Data S7 **Numerical data for the FSC plot in Figure S5C.**
(XLSX)

Data S8 **Numerical data for the rotational correlation plot in Figure S8A.**
(XLSX)

Data S9 **Numerical data for the rotational correlation plot in Figure S8B.**
(XLSX)

Movie S1 **Membrane-containing phage PRD1 cryo-EM reconstruction.**
(MOV)

Movie S2 **Delineating the boundary between the unique vertex complex and its surrounding capsid proteins.**
(MOV)

Movie S3 **Membrane-containing phage PRD1 procapsid cryo-EM reconstruction.**
(MOV)

## Abbreviations

cryo-EM: electron cryo-microscopy
dsDNA: double-stranded DNA
FSC: Fourier shell correlation
MCP: major capsid protein
MPSA: multi-path simulated annealing
WT: wild-type
