# Supplementary figures and images for: A Structural Model of the Genome Packaging Process in a Membrane-Containing Double Stranded DNA Virus
Source: PLoS Biol. 2014 Dec 16;12(12):e1002024. doi: 10.1371/journal.pbio.1002024 (PMC4267777; doi:10.1371/journal.pbio.1002024)

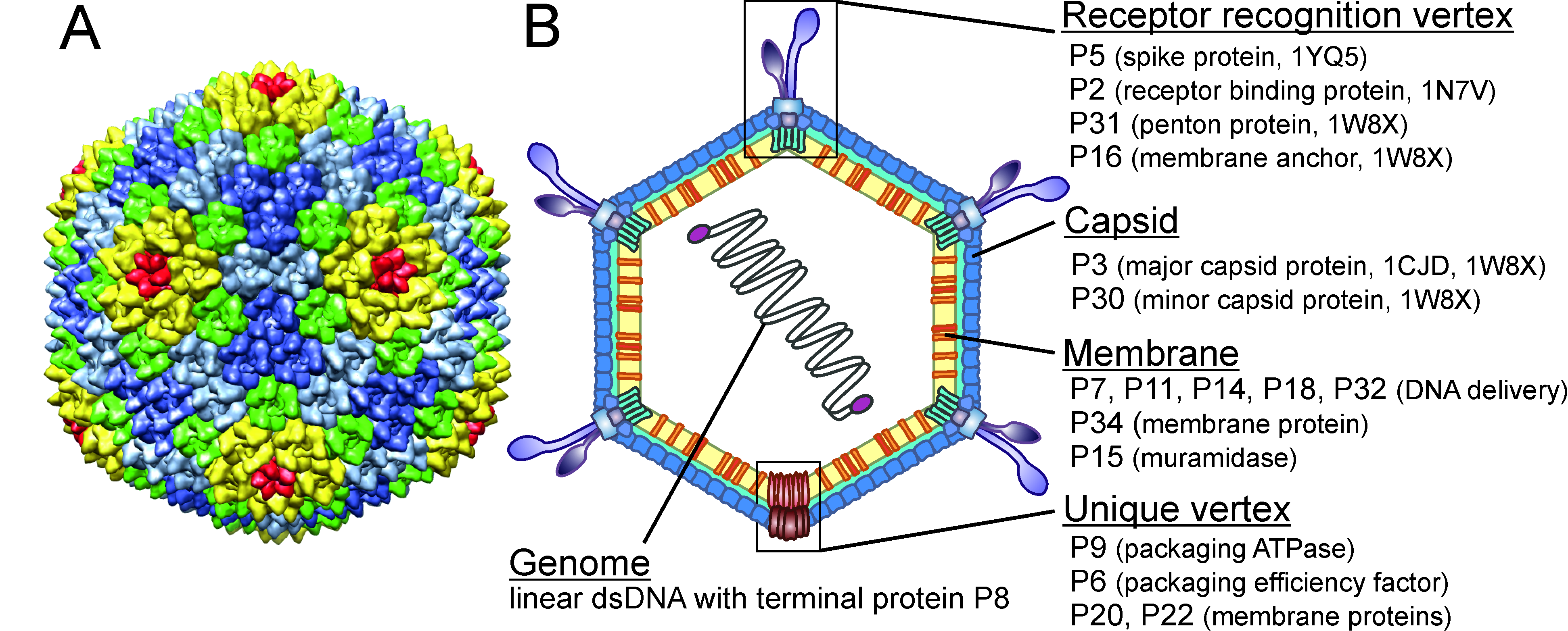

Supplement: Figure S1 — Schematic illustration of PRD1 virion. (A) PRD1 capsomer organization (PDB 1W8X) visualized by Chimera. Four MCP P3 trimers forming the asymmetric unit are colored in green, light blue, blue, and yellow. The penton protein P31 at the vertices is in red. (B) A schematic presentation of the PRD1 virion and functions of the virion proteins. Numbers in the parenthesis identify the corresponding protein structures in the Protein Data Bank. (TIF) [file pbio.1002024.s001.tif]

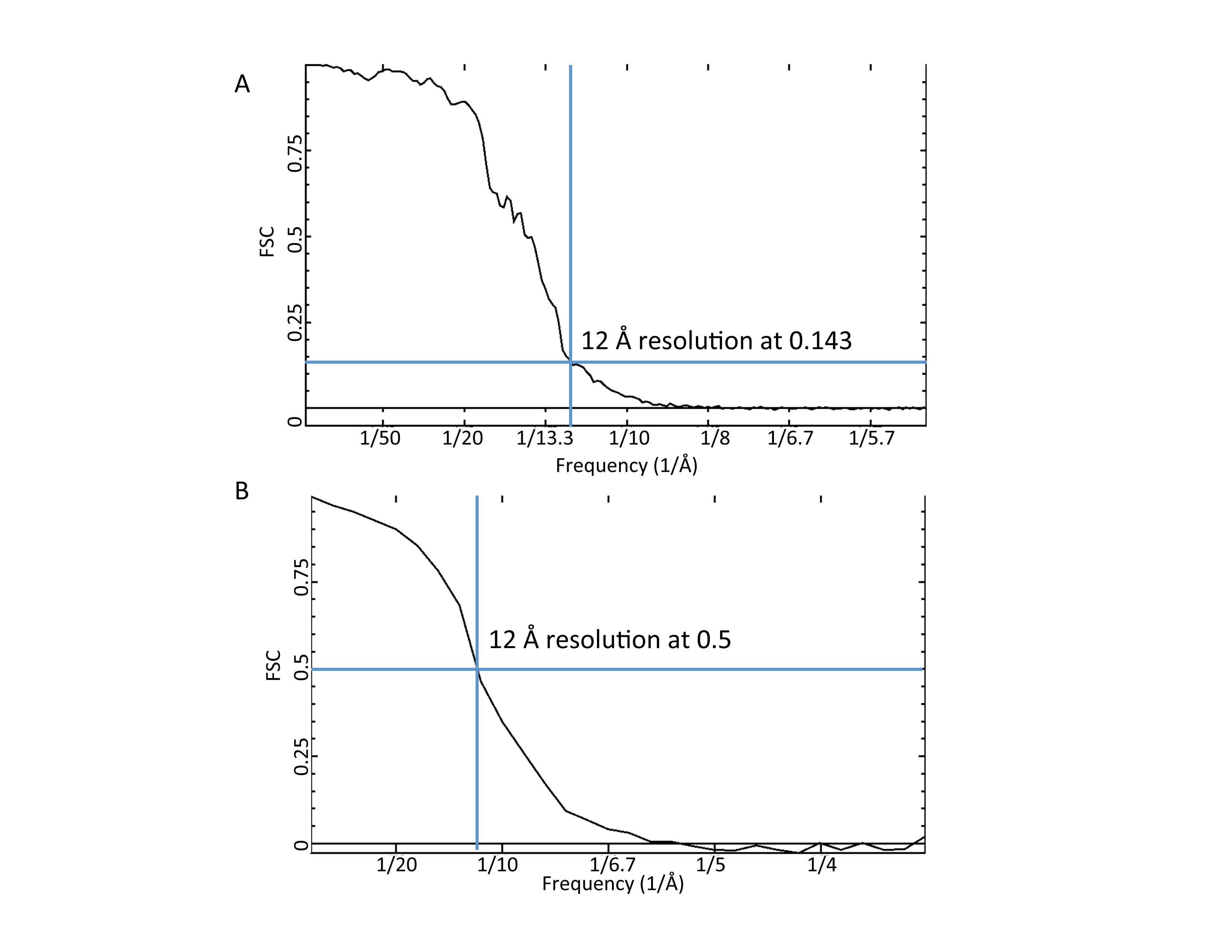

Supplement: Figure S2 — Resolution assessment for reconstruction of the virion without icosahedral symmetry imposition. (A) FSC curve of gold-standard resolution test of the reconstruction without icosahedral symmetry imposition and without masking reveals the resolution to be 12 Å at 0.143 criterion. (B) Calculated FSC curve between the X-ray structure of P3 (PDB code: 1W8X, chain B) to the segmented density of P3 from our cryo-EM map reveals the resolution to be 12 Å at 0.5 criterion. (TIF) [file pbio.1002024.s002.tif]

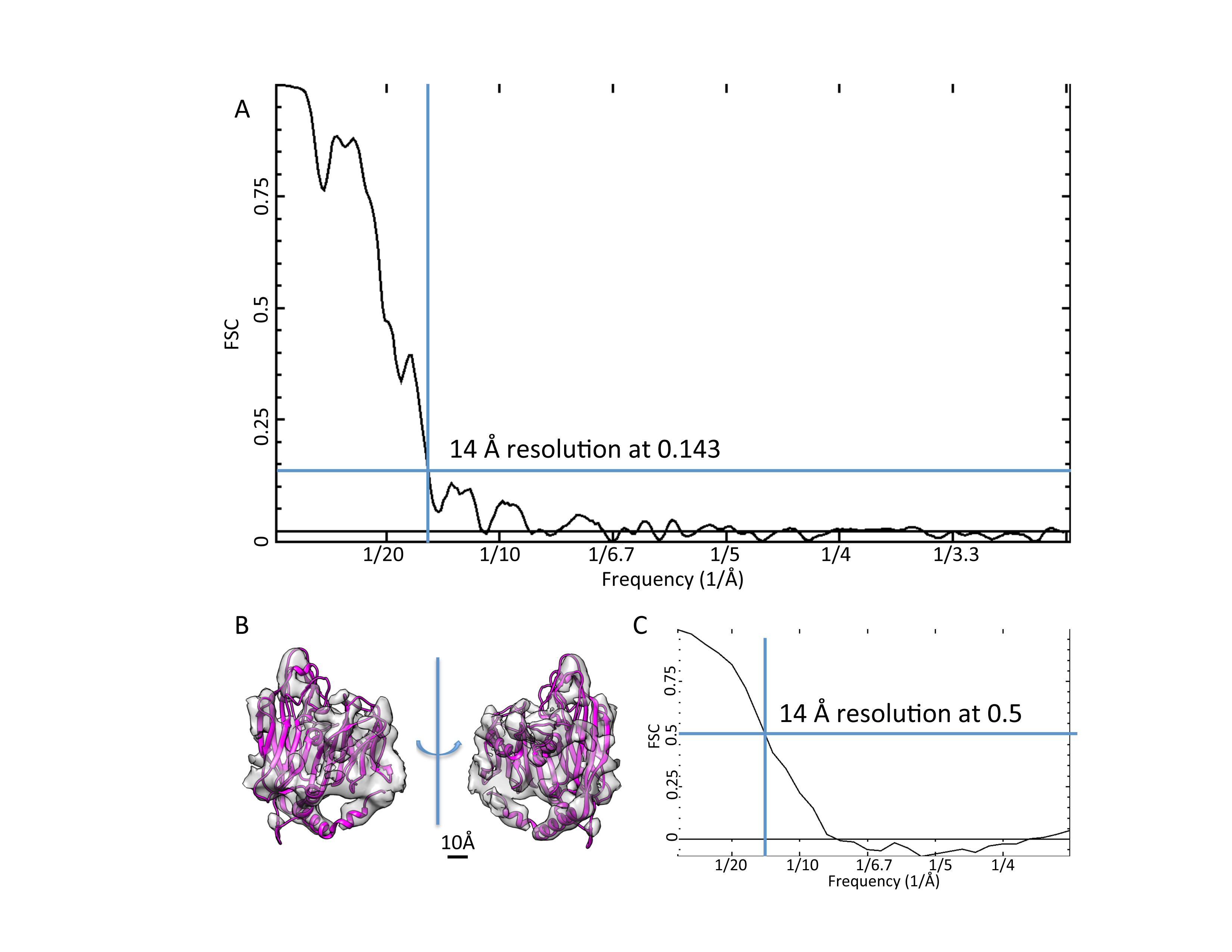

Supplement: Figure S3 — Resolution assessment for reconstruction of the procapsid without icosahedral symmetry imposition. (A) FSC curve of gold-standard resolution test of the reconstruction without icosahedral symmetry imposition and without masking reveals the resolution to be 14 Å at 0.143 criterion. (B) Segmented density of P3 chain B from the cryo-EM map of the procapsid docking with the corresponding crystal structure (PDB code: 1W8X, chain B). (C) Calculated FSC curve between the X-ray structure of P3 to our cryo-EM map reveals the resolution to be 14 Å at 0.5 criterion. (TIF) [file pbio.1002024.s003.tif]

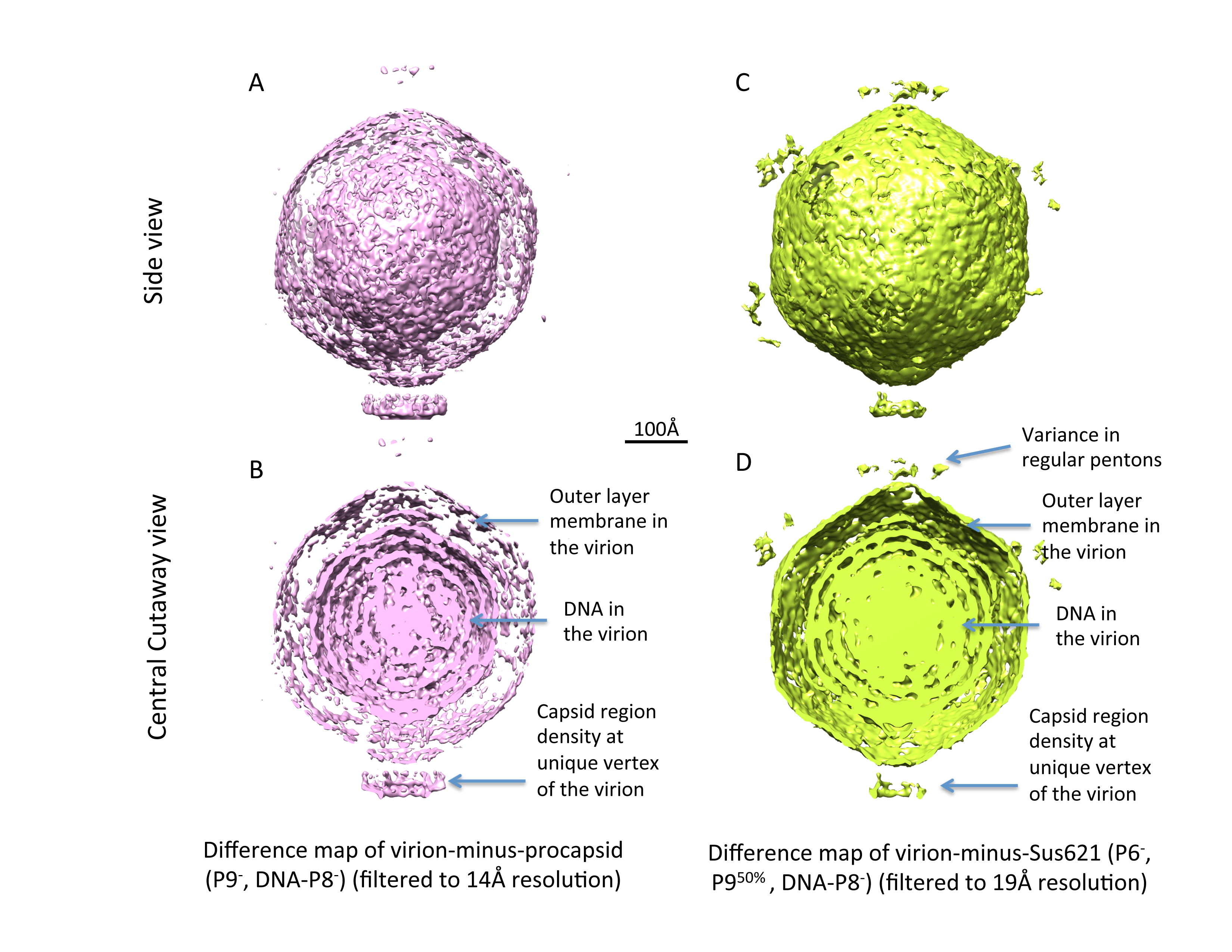

Supplement: Figure S4 — Difference maps of procapsid (P9−, DNA-P8−) and Sus621 mutant particle (P6−, P950%, DNA-P8−) compared to the mature virion. (A) The side view and (B) the central cutaway view of the difference map between the procapsid and the virion filtered to the same 14 Å resolution. (C) The side view and (D) the central cutaway view of the difference map between the Sus621 mutant particle and the virion filtered to the same 19 Å resolution. (TIF) [file pbio.1002024.s004.tif]

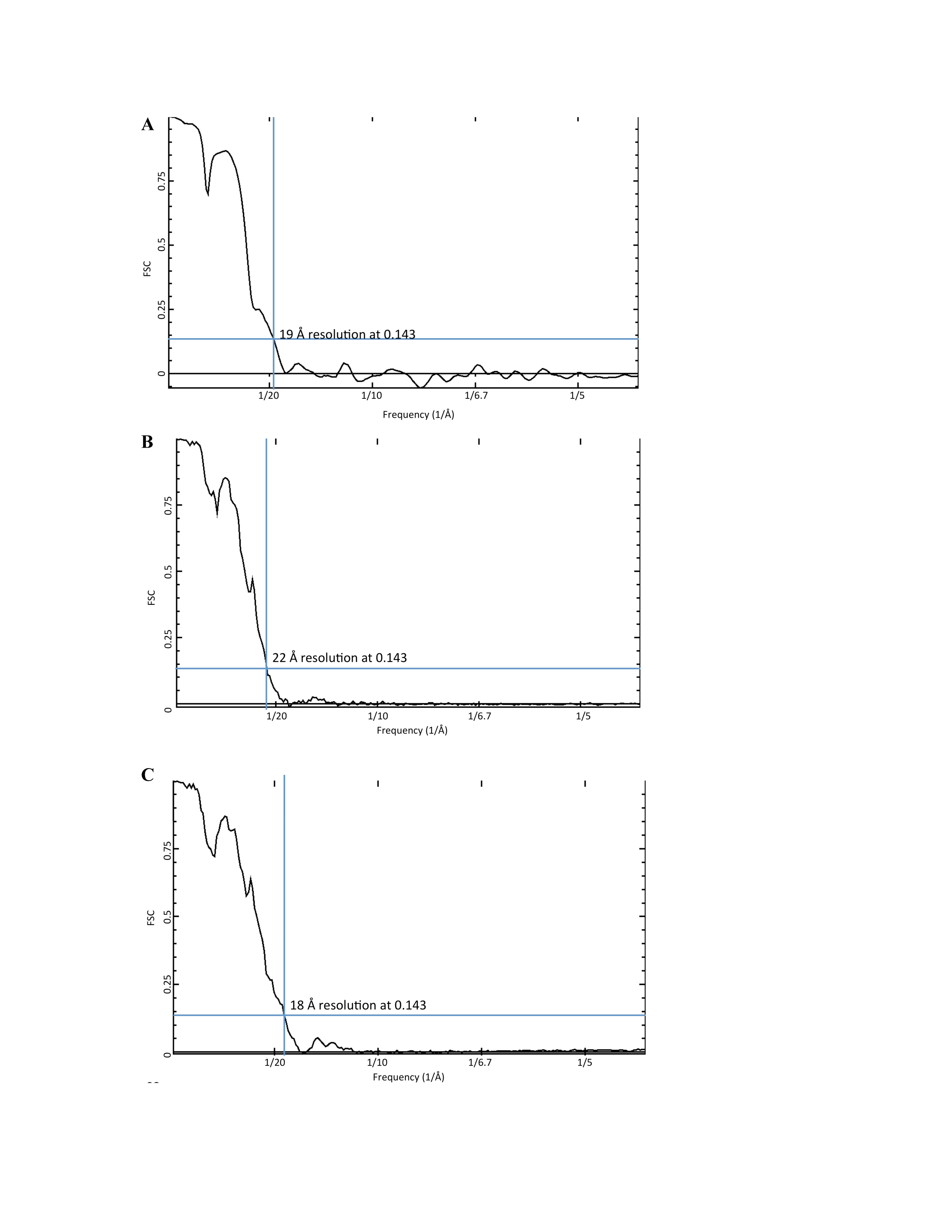

Supplement: Figure S5 — Resolution assessments for the reconstruction of the Sus621, Sus526, and Sus42 mutant particles without icosahedral symmetry imposition. (A) FSC curves of gold-standard resolution test of the Sus621 particle reconstruction without icosahedral symmetry imposition and without masking reveals the resolution to be 19 Å at 0.143 criterion. (B) FSC curves of gold-standard resolution test of the Sus526 particle reconstruction without icosahedral symmetry imposition and without masking reveals the resolution to be 22 Å at 0.143 criterion. (C) FSC curves of gold-standard resolution test of the Sus42 particle reconstruction without icosahedral symmetry imposition and without masking reveals the resolution to be 18 Å at 0.143 criterion. (TIF) [file pbio.1002024.s005.tif]

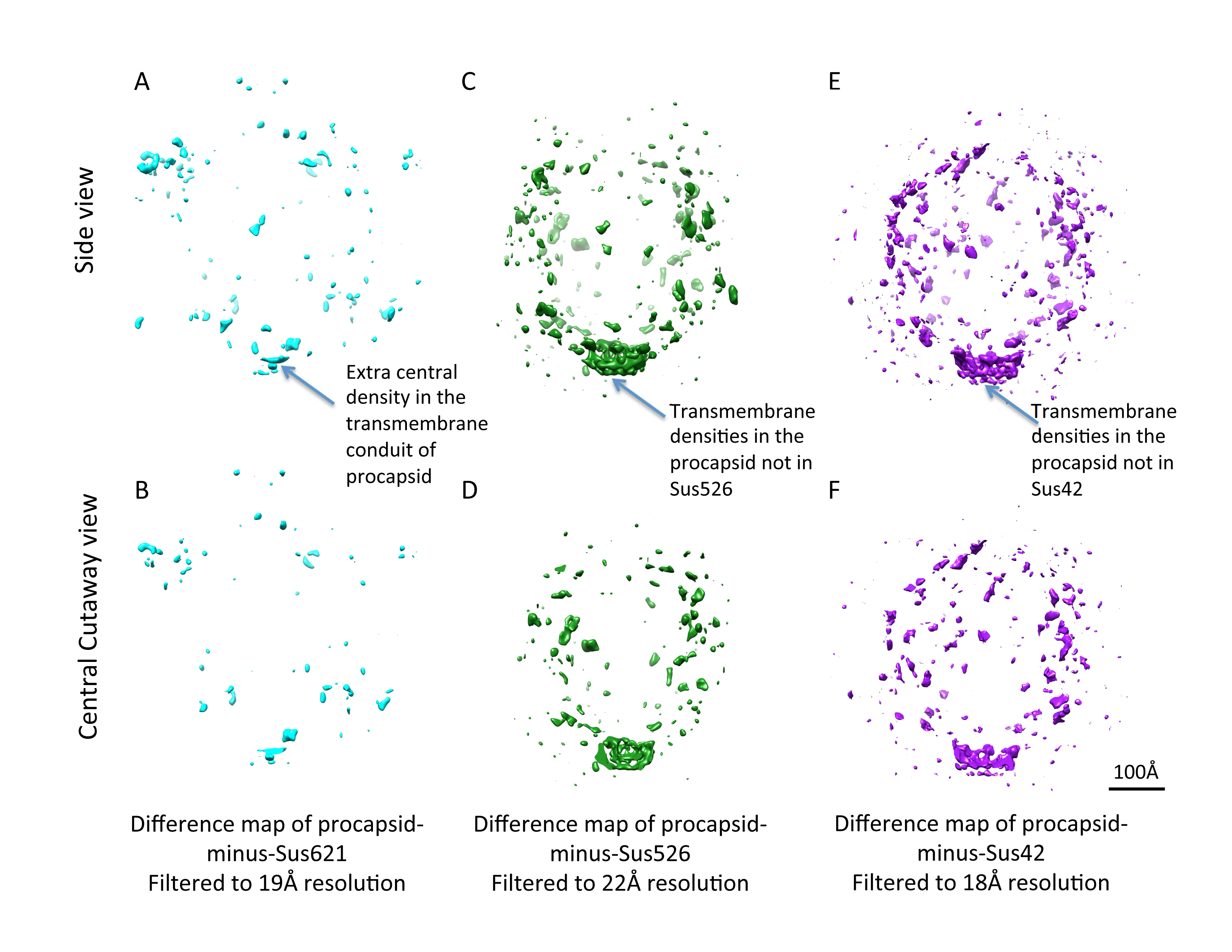

Supplement: Figure S6 — Difference maps of the Sus621 (P6−, P950%, DNA-P8−), Sus526 (P6−, P9−, P20− [P22−], DNA-P8−) and Sus42 (P6−, P9−, [P20−], P22−, DNA-P8−) particles to the procapsid (P9−, DNA-P8−). (A) The side view and (B) the central cutaway view of the difference map between the Sus621 and the procapsid filtered to the same 19 Å resolution. (C) The side view and (D) the central cutaway view of the difference map between the Sus526 particle and the procapsid filtered to the same 22 Å resolution. (E) The side view and (F) the central cutaway view of the difference map between the Sus42 particle and the procapsid filtered to the same 18 Å resolution. (TIF) [file pbio.1002024.s006.tif]

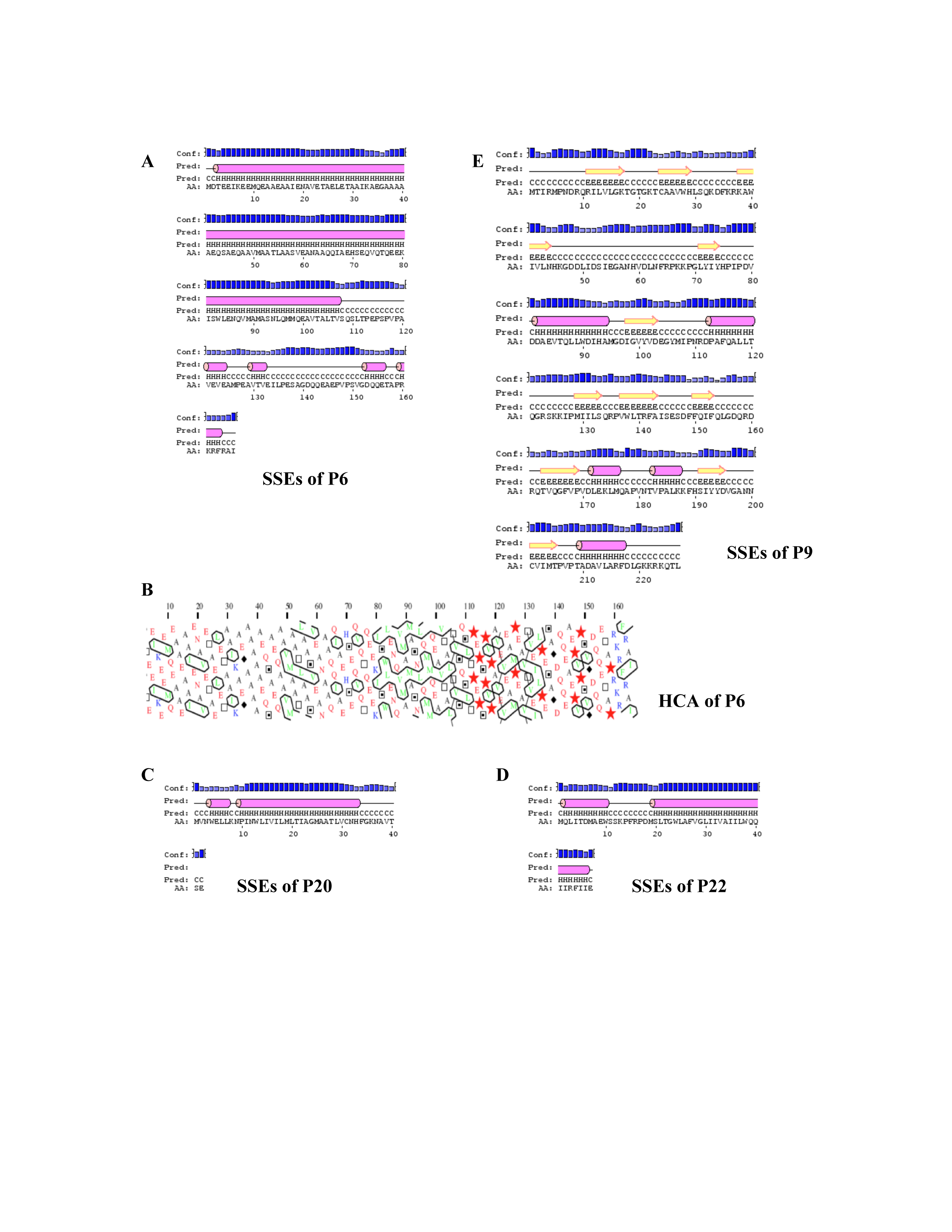

Supplement: Figure S7 — Secondary structural elements predictions and hydrophobicity cluster analysis. (A) Secondary structural elements (SSEs) prediction by psipred for P6. (B) Hydrophobicity cluster analysis by HCA for P6. (C) SSEs prediction for P20. (D) SSEs prediction for P22. (E) SSEs prediction for P9. (TIF) [file pbio.1002024.s007.tif]

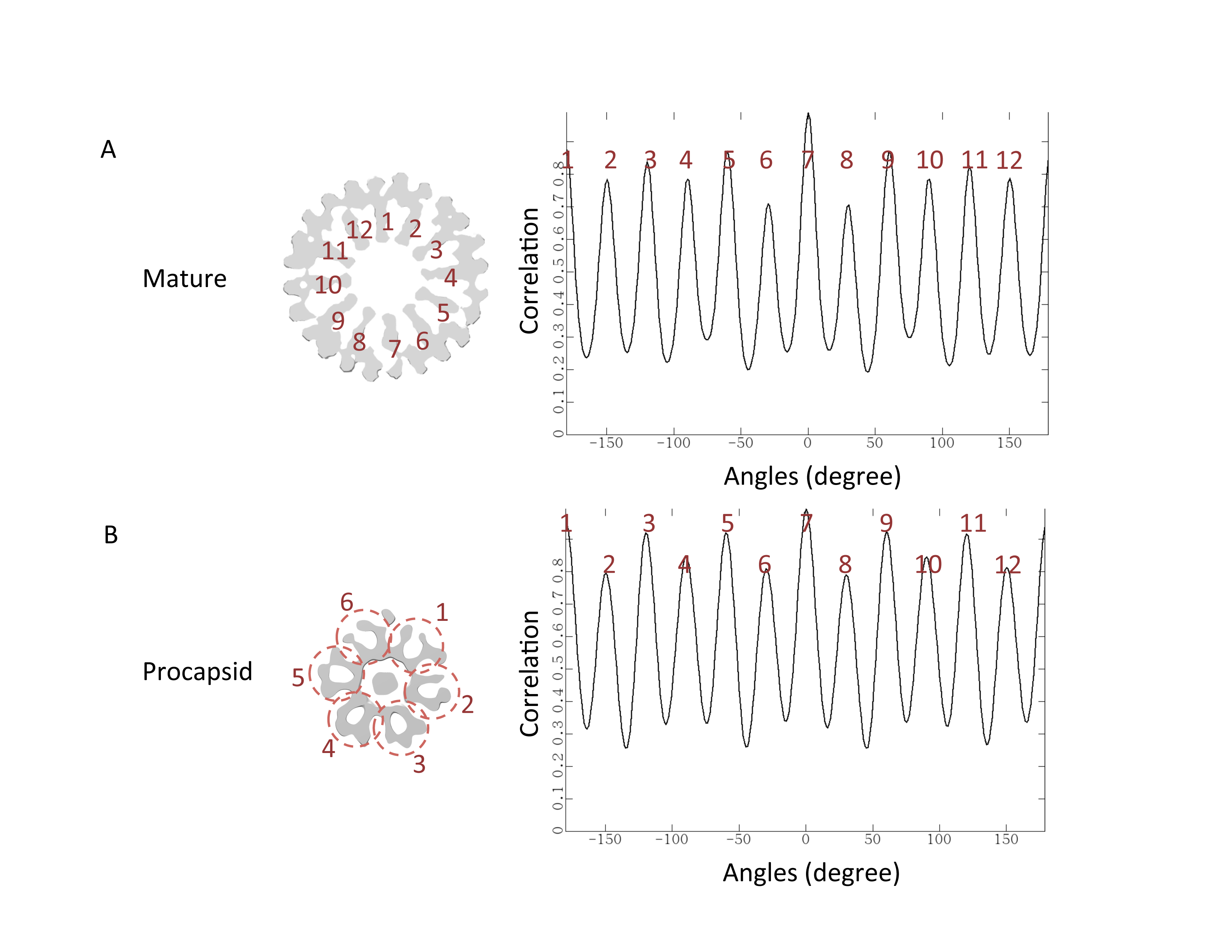

Supplement: Figure S8 — Rotational correlation analysis. (A) Rotational correlation curve of the unique vertex in the mature virus and (B) Rotational correlation curve of the transmembrane densities in the procapsid. (TIF) [file pbio.1002024.s008.tif]

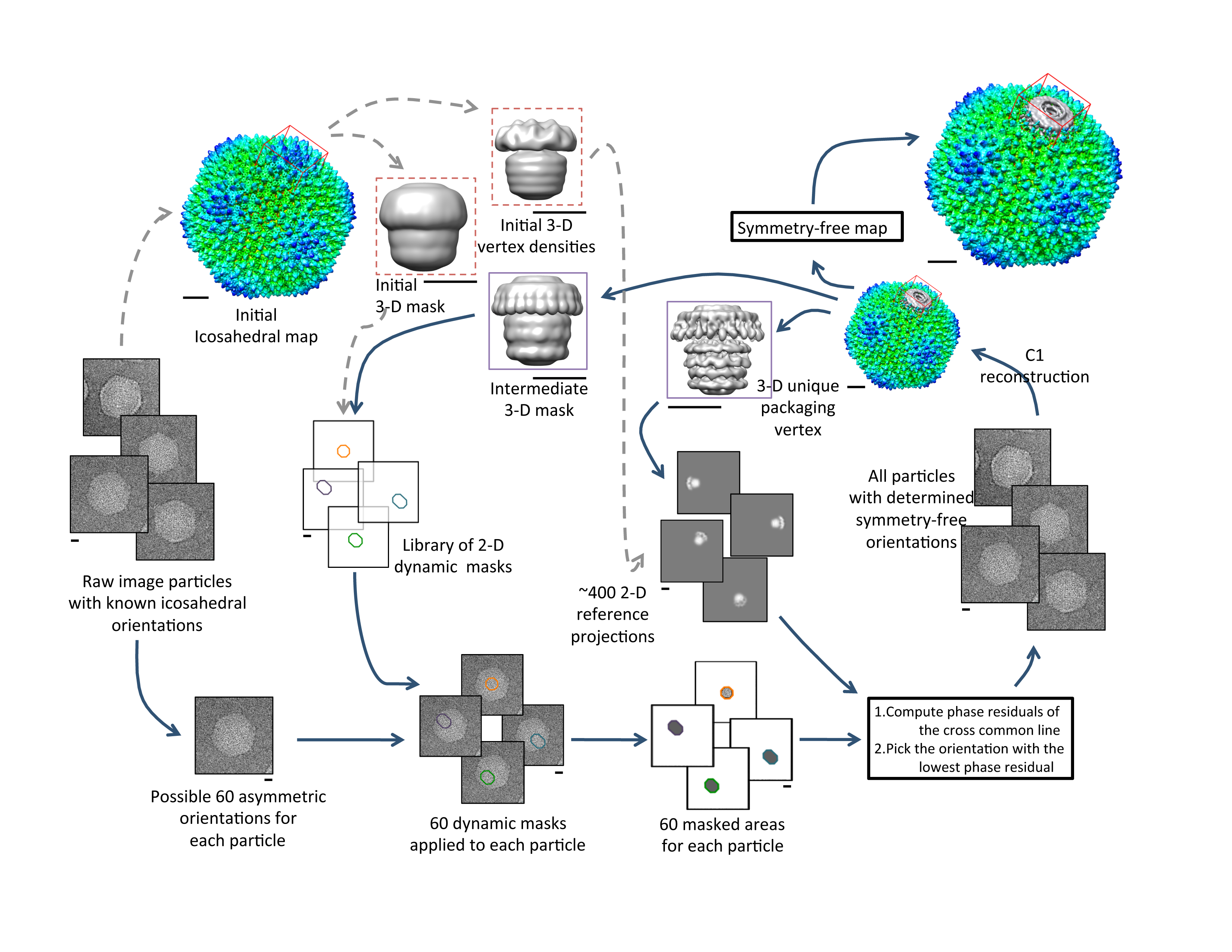

Supplement: Figure S9 — An illustration for the workflow of virus reconstruction without icosahedral symmetry imposition. Using the raw particle images, the icosahedral orientations were first determined to reconstruct the icosahedral map. An initial vertex volume (in the red cubic area) was extracted from the icosahedral map and made into a 3-D initial model as a mask of a putative portal. This initial 3-D portal mask was used to generate a library of 2-D masks corresponding to 60 possible locations for each raw particle image with known icosahedral orientation. 60 corresponding masked areas were extracted from each particle image and compared using cross common lines to the projections of the initial mask model. The best match among all the comparisons represents the most likely location of the unique vertex. The orientation that corresponds to the best match in the search for the unique vertex is the true asymmetric orientation for the particle. 3-D map of the virus would be reconstructed from the particles with true asymmetric orientations without imposing any symmetry. This process was iterated until the map had converged and no improvements could be seen. (TIF) [file pbio.1002024.s009.tif]
