# Supplementary material for: A Structural Model of the Genome Packaging Process in a Membrane-Containing Double Stranded DNA Virus
Source: PLoS Biol. 2014 Dec 16;12(12):e1002024. doi: 10.1371/journal.pbio.1002024 (PMC4267777; doi:10.1371/journal.pbio.1002024)
Supplement: Table S1 — Escherichia coli K12 HMS174 strains and plasmids used in the complementation assay. (DOCX) [file pbio.1002024.s010.docx]

**Table S1.** *Escherichia coli* K12 HMS174 strains and plasmids used in the complementation assay [[1](#_ENREF_1)]

| **PRD1 mutant** | **Complementation control strain** | **Complementation strain** |
| --- | --- | --- |
| *sus621* | HMS174(DE3)(pLM2)(pJJ2) | HMS174(DE3)(pLM2)(pNS62) |
| *sus42* | HMS174(pLM2)(pSU18) | HMS174(pLM2)(pMV11) |
| *sus526* | HMS174(pLM2)(pSU18) | HMS174(pLM2)(pMG119) |
| **Plasmid** | **Description** | **Reference** |
| pLM2 | encodes the PRD1 receptor, IncPα, Km^r^ | [[2](#_ENREF_2)] |
| pJJ2 | expression vertor, ColE1, Ap^r^ | [[3](#_ENREF_3)] |
| pSU18 | cloning vertor, p15A, Cm^r^ | [[4](#_ENREF_4)] |
| pNS62 | PRD1 gene *VI* in pJJ2 | [[5](#_ENREF_5)] |
| pMV11 | PRD1 gene *XXII* in pSU18 | [[6](#_ENREF_6)] |
| pMG119 | PRD1 gene *XX* in pSU18 | [[6](#_ENREF_6)] |

References:

1. Campbell JL, Richardson CC, Studier FW (1978) Genetic recombination and complementation between bacteriophage T7 and cloned fragments of T7 DNA. Proc Natl Acad Sci U S A 75: 2276-2280.

2. Mindich L, Cohen J, Weisburd M (1976) Isolation of nonsense suppressor mutants in Pseudomonas. J Bacteriol 126: 177-182.

3. Ojala PM, Juuti JT, Bamford DH (1993) Protein P4 of double-stranded RNA bacteriophage phi 6 is accessible on the nucleocapsid surface: epitope mapping and orientation of the protein. J Virol 67: 2879-2886.

4. Bartolome B, Jubete Y, Martinez E, de la Cruz F (1991) Construction and properties of a family of pACYC184-derived cloning vectors compatible with pBR322 and its derivatives. Gene 102: 75-78.

5. Karhu NJ, Ziedaite G, Bamford DH, Bamford JK (2007) Efficient DNA packaging of bacteriophage PRD1 requires the unique vertex protein P6. J Virol 81: 2970-2979.

6. Strömsten NJ, Bamford DH, Bamford JK (2003) The unique vertex of bacterial virus PRD1 is connected to the viral internal membrane. J Virol 77: 6314-6321.
