# Supplementary material for: A Structural Model of the Genome Packaging Process in a Membrane-Containing Double Stranded DNA Virus
Source: PLoS Biol. 2014 Dec 16;12(12):e1002024. doi: 10.1371/journal.pbio.1002024 (PMC4267777; doi:10.1371/journal.pbio.1002024)
Supplement: Table S2 — Data collection of the virion, the procapsid, and three packaging mutant particles. (DOCX) [file pbio.1002024.s011.docx]

**Table S2.** Data collection of the virion, the procapsid and three packaging mutants.

| Samples^1^ | Imaging conditions | Number of CCD frames | Number of boxed particles | Number of particles in final map | Gold-standard FSC resolution test | | |
| --- | --- | --- | --- | --- | --- | --- | --- |
|  |  |  |  |  | Number of particles  Odd Half | Number of particles  Even Half | Resolutions |
| Mature virion | Dataset1: JEM3200FSC; 1.42 Å/pixel; 4K CCD  Dataset2: JEM3200FSC; 1.3 Å/pixel; 10K CCD binned by 2 | Dataset1: 564 frames  Dataset2:  880 frames | Dataset1: 13,000  Dataset2:  37,000  Total: 50,000 | Total:  26,000 | 12,400  out of  25,000 | 12,900  out of  25,000 | 12Å |
| Procapsid | JEM3200FSC; 1.42Å/pixel; 4K CCD | 340 frames | 5,203 | 4,300 | 2,070  out of  2,601 | 2,200  out of  2,601 | 14Å |
| Sus621 | JEM2010F; 2.18Å/pixel; 4K CCD | 122 frames | 3,300 | 2,800 | 1,460  out of  1,650 | 1480  out of  1,650 | 19Å |
| Sus526 | JEM2010F; 2.18Å/pixel; 4K CCD | 117 frames | 4,063 | 3,600 | 1820  out of  2,031 | 1840  out of  2,031 | 22Å |
| Sus42 | JEM2010F; 2.18Å/pixel; 4K CCD | 116 frames | 3,496 | 3,000 | 1,630  out of  1,748 | 1580  out of  1,748 | 18Å |

^1^ See Table 1
